# 3-Methyl­sulfinyl-2-phenyl-1-benzofuran

**DOI:** 10.1107/S1600536808024276

**Published:** 2008-08-06

**Authors:** Hong Dae Choi, Pil Ja Seo, Byeng Wha Son, Uk Lee

**Affiliations:** a Department of Chemistry, Dongeui University, San 24 Kaya-dong, Busanjin-gu, Busan 614-714, Republic of Korea; b Department of Chemistry, Pukyong National University, 599-1 Daeyeon 3-dong, Nam-gu, Busan 608-737, Republic of Korea

## Abstract

The title compound, C_15_H_12_O_2_S, was prepared by the oxidation of 3-methyl­sulfanyl-2-phenyl-1-benzofuran with 3-chloro­peroxy­benzoic acid. The phenyl ring makes a dihedral angle of 37.65 (8)° with the plane of the benzofuran fragment. The O atom and the methyl group of the methyl­sulfinyl substituent lie on opposite sides of the plane of the benzofuran ring system. The crystal structure is stabilized by aromatic π–π inter­actions between the benzene rings of neighbouring mol­ecules [centroid–centroid distance = 3.549 (2) Å] and by inter­molecular C—H⋯O inter­actions.

## Related literature

For the crystal structures of similar 3-methyl­sulfinyl-2-phenyl-1-benzofuran compounds, see: Choi *et al.* (2007*a* ▶,*b* ▶).
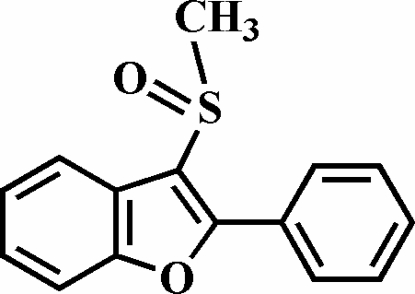


## Experimental

### 

#### Crystal data

C_15_H_12_O_2_S
*M* *_r_* = 256.32
Triclinic, 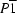

*a* = 8.0185 (8) Å
*b* = 9.4381 (9) Å
*c* = 9.7749 (9) Å
α = 115.574 (2)°
β = 109.179 (2)°
γ = 94.296 (2)°
*V* = 609.51 (10) Å^3^
*Z* = 2
Mo *K*α radiation
μ = 0.26 mm^−1^
*T* = 173 (2) K
0.30 × 0.10 × 0.10 mm

#### Data collection

Bruker SMART CCD diffractometer
Absorption correction: none
3185 measured reflections
2120 independent reflections
1878 reflections with *I* > 2σ(*I*)
*R*_int_ = 0.030

#### Refinement

*R*[*F* ^2^ > 2σ(*F* ^2^)] = 0.037
*wR*(*F* ^2^) = 0.090
*S* = 1.10
2120 reflections
164 parameters
H-atom parameters constrained
Δρ_max_ = 0.32 e Å^−3^
Δρ_min_ = −0.24 e Å^−3^

### 

Data collection: *SMART* (Bruker, 2001 ▶); cell refinement: *SAINT* (Bruker, 2001 ▶); data reduction: *SAINT*; program(s) used to solve structure: *SHELXS97* (Sheldrick, 2008 ▶); program(s) used to refine structure: *SHELXL97* (Sheldrick, 2008 ▶); molecular graphics: *ORTEP-3* (Farrugia, 1997 ▶) and *DIAMOND* (Brandenburg, 1998 ▶); software used to prepare material for publication: *SHELXL97*.

## Supplementary Material

Crystal structure: contains datablocks global, I. DOI: 10.1107/S1600536808024276/pk2109sup1.cif

Structure factors: contains datablocks I. DOI: 10.1107/S1600536808024276/pk2109Isup2.hkl

Additional supplementary materials:  crystallographic information; 3D view; checkCIF report

## Figures and Tables

**Table 1:** Hydrogen-bond geometry (Å, °)

*D*—H⋯*A*	*D*—H	H⋯*A*	*D*⋯*A*	*D*—H⋯*A*
C15—H15*C*⋯O2^i^	0.98	2.34	3.290 (3)	164

Symmetry code: (i) 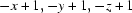.

**Fig. 1.**
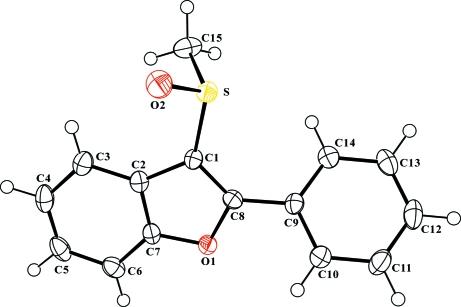
The molecular structure of the title compound, showing displacement ellipsoids drawn at the 50% probability level.

**Fig. 2.**
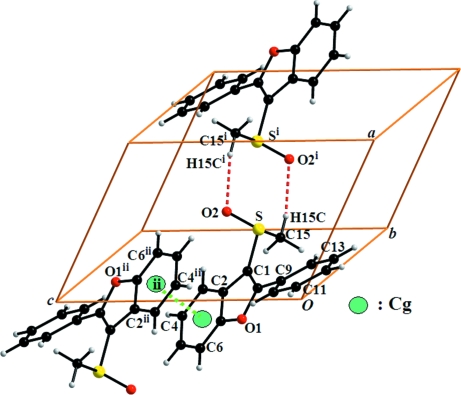
π—π and C—H···O interactions (dotted lines) in the title compound. Cg denotes ring centroid. [Symmetry code: (i) -x + 1, -y + 1, -z + 1; (ii) -x, -y, -z + 1.]

## supplementary crystallographic information

### Comment

This work is related to our previous communications on the synthesis and structure of 3-methylsulfinyl-2-phenyl-1-benzofuran analogues, *viz*. 5-chloro-3-methylsulfinyl-2-phenyl-1-benzofuran (Choi *et al.*, 2007*a*) and 5-methyl-3-methylsulfinyl-2-phenyl-1-benzofuran (Choi *et al.*, 2007*b*). Here we report the crystal structure of 3-methylsulfinyl-2-phenyl-1-benzofuran (Fig. 1).

The benzofuran unit is essentially planar, with a mean deviation of 0.009 (2) Å from the least-squares plane defined by the nine constituent atoms. The phenyl ring (C9—C14) makes a dihedral angle of 37.65 (8)° with the plane of the benzofuran fragment. The molecular packing (Fig. 2) is stabilized by aromatic π—π stacking interactions between the benzene rings from the adjacent molecules. The *Cg*···*Cg*^ii^ distance is 3.549 (2) Å (*Cg* is the centroid of C2—C7 benzene ring, symmetry code as in Fig. 2). The crystal structure is further stabilized by C—H···O  (Fig. 2) interactions between a methyl H atom and the oxygen of the S=O unit, with a C15—H15C···O2^i^ separation of 2.36 Å (Fig. 2 and Table 1; symmetry code as in Fig. 2).

### Experimental

77% 3-Chloroperoxybenzoic acid (359 mg, 1.6 mmol) was added in small portions to a stirred solution of 3-methylsulfanyl-2-phenyl-1-benzofuran (360 mg, 1.5 mmol) in dichloromethane (30 ml) at 273 K. After being stirred for 2 h at room temperature, the mixture was washed with saturated sodium bicarbonate solution and the organic layer was separated, dried over magnesium sulfate, filtered and concentrated under vacuum. The residue was purified by column chromatography (hexane-ethyl acetate, 1: 2 *v*/*v*) to afford the title compound as a colorless solid [yield 76%, m.p. 408–409 K; *R*_f_ = 0.79 (hexane-ethyl acetate, 1:2 *v*/*v*)]. Single crystals suitable for X-ray diffraction were prepared by evaporation of a solution of the title compound in benzene at room temperature. Spectroscopic analysis: ^1^H NMR (CDCl_3_, 400 MHz) δ 3.13 (s, 3H), 7.33–7.44 (m, 3H), 7.48–7.54 (m, 2H), 7.59 (d, J = 8.03 Hz, 1H), 7.84 (dd, J = 8.08 Hz and J = 1.48 Hz, 2H), 8.22 (d, J = 7.32 Hz, 1H); EI—MS 256 [*M*^+^].

### Refinement

All H atoms were geometrically positioned and refined using a riding model, with C—H = 0.95 Å for aromatic H atoms, 0.98 Å for methyl H atoms, respectively, and with *U*_iso_(H) = 1.2Ueq(C) for aromatic H atoms and 1.5Ueq(C) for methyl H atoms.

### Crystal data

**Table:**

C_15_H_12_O_2_S	*Z* = 2
*M**_r_* = 256.32	*F*_000_ = 268
Triclinic, *P*1	*D*_x_ = 1.397 Mg m^−^^3^
Hall symbol: -P 1	Mo *K*α radiation λ = 0.71073 Å
*a* = 8.0185 (8) Å	Cell parameters from 2383 reflections
*b* = 9.4381 (9) Å	θ = 2.5–28.2º
*c* = 9.7749 (9) Å	µ = 0.26 mm^−^^1^
α = 115.574 (2)º	*T* = 173 (2) K
β = 109.179 (2)º	Block, colorless
γ = 94.296 (2)º	0.30 × 0.10 × 0.10 mm
*V* = 609.51 (10) Å^3^	

### Data collection

**Table:**

Bruker SMART CCD diffractometer	2120 independent reflections
Radiation source: fine-focus sealed tube	1878 reflections with *I* > 2σ(*I*)
Monochromator: graphite	*R*_int_ = 0.030
Detector resolution: 10.0 pixels mm^-1^	θ_max_ = 25.0º
*T* = 173(2) K	θ_min_ = 2.5º
φ and ω scans	*h* = −8→9
Absorption correction: none	*k* = −11→11
3185 measured reflections	*l* = −11→5

### Refinement

**Table:**

Refinement on *F*^2^	Secondary atom site location: difference Fourier map
Least-squares matrix: full	Hydrogen site location: inferred from neighbouring sites
*R*[*F*^2^ > 2σ(*F*^2^)] = 0.037	H-atom parameters constrained
*wR*(*F*^2^) = 0.090	*w* = 1/[σ^2^(*F*_o_^2^) + (0.0341*P*)^2^ + 0.3746*P*] where *P* = (*F*_o_^2^ + 2*F*_c_^2^)/3
*S* = 1.10	(Δ/σ)_max_ < 0.001
2120 reflections	Δρ_max_ = 0.32 e Å^−^^3^
164 parameters	Δρ_min_ = −0.24 e Å^−^^3^
Primary atom site location: structure-invariant direct methods	Extinction correction: none

### Special details

**Table:**

Geometry. All e.s.d.'s (except the e.s.d. in the dihedral angle between two l.s. planes) are estimated using the full covariance matrix. The cell e.s.d.'s are taken into account individually in the estimation of e.s.d.'s in distances, angles and torsion angles; correlations between e.s.d.'s in cell parameters are only used when they are defined by crystal symmetry. An approximate (isotropic) treatment of cell e.s.d.'s is used for estimating e.s.d.'s involving l.s. planes.
Refinement. Refinement of *F*^2^ against ALL reflections. The weighted *R*-factor *wR* and goodness of fit *S* are based on *F*^2^, conventional *R*-factors *R* are based on *F*, with *F* set to zero for negative *F*^2^. The threshold expression of *F*^2^ > σ(*F*^2^) is used only for calculating *R*-factors(gt) *etc*. and is not relevant to the choice of reflections for refinement. *R*-factors based on *F*^2^ are statistically about twice as large as those based on *F*, and *R*- factors based on ALL data will be even larger.

### Fractional atomic coordinates and isotropic or equivalent isotropic displacement parameters (Å^2^)

**Table:**

	*x*	*y*	*z*	*U*_iso_*/*U*_eq_	
S	0.33893 (6)	0.23694 (6)	0.35764 (6)	0.02368 (16)	
O1	−0.05250 (18)	−0.15771 (15)	0.17293 (16)	0.0238 (3)	
O2	0.4116 (2)	0.33041 (18)	0.54205 (18)	0.0356 (4)	
C1	0.1412 (3)	0.0880 (2)	0.2927 (2)	0.0210 (4)	
C2	0.0021 (3)	0.1096 (2)	0.3590 (2)	0.0219 (4)	
C3	−0.0353 (3)	0.2390 (3)	0.4755 (3)	0.0280 (5)	
H3	0.0420	0.3457	0.5336	0.034*	
C4	−0.1883 (3)	0.2063 (3)	0.5031 (3)	0.0321 (5)	
H4	−0.2170	0.2928	0.5807	0.039*	
C5	−0.3018 (3)	0.0500 (3)	0.4202 (3)	0.0326 (5)	
H5	−0.4060	0.0329	0.4425	0.039*	
C6	−0.2666 (3)	−0.0807 (3)	0.3064 (3)	0.0291 (5)	
H6	−0.3429	−0.1877	0.2500	0.035*	
C7	−0.1131 (3)	−0.0453 (2)	0.2802 (2)	0.0226 (4)	
C8	0.1044 (2)	−0.0725 (2)	0.1847 (2)	0.0212 (4)	
C9	0.1918 (3)	−0.1716 (2)	0.0813 (2)	0.0221 (4)	
C10	0.1916 (3)	−0.3292 (2)	0.0565 (3)	0.0299 (5)	
H10	0.1393	−0.3695	0.1110	0.036*	
C11	0.2669 (3)	−0.4265 (3)	−0.0468 (3)	0.0370 (5)	
H11	0.2666	−0.5331	−0.0624	0.044*	
C12	0.3428 (3)	−0.3693 (3)	−0.1276 (3)	0.0364 (5)	
H12	0.3931	−0.4370	−0.1995	0.044*	
C13	0.3451 (3)	−0.2136 (3)	−0.1033 (3)	0.0315 (5)	
H13	0.3986	−0.1738	−0.1575	0.038*	
C14	0.2695 (3)	−0.1150 (2)	0.0000 (2)	0.0255 (4)	
H14	0.2707	−0.0083	0.0154	0.031*	
C15	0.2300 (3)	0.3619 (3)	0.2805 (3)	0.0352 (5)	
H15A	0.1334	0.3896	0.3203	0.053*	
H15B	0.1768	0.3027	0.1592	0.053*	
H15C	0.3203	0.4616	0.3200	0.053*	

### Atomic displacement parameters (Å^2^)

**Table:**

	*U*^11^	*U*^22^	*U*^33^	*U*^12^	*U*^13^	*U*^23^
S	0.0189 (3)	0.0221 (3)	0.0235 (3)	0.00303 (19)	0.0068 (2)	0.0072 (2)
O1	0.0233 (7)	0.0214 (7)	0.0253 (7)	0.0042 (5)	0.0117 (6)	0.0089 (6)
O2	0.0311 (8)	0.0359 (9)	0.0232 (8)	−0.0025 (7)	0.0057 (7)	0.0058 (7)
C1	0.0201 (10)	0.0212 (10)	0.0202 (10)	0.0065 (8)	0.0080 (8)	0.0088 (8)
C2	0.0204 (10)	0.0264 (10)	0.0206 (10)	0.0093 (8)	0.0080 (8)	0.0122 (9)
C3	0.0305 (11)	0.0281 (11)	0.0246 (11)	0.0125 (9)	0.0107 (9)	0.0116 (9)
C4	0.0357 (12)	0.0421 (13)	0.0277 (11)	0.0238 (10)	0.0181 (10)	0.0185 (10)
C5	0.0303 (12)	0.0506 (14)	0.0401 (13)	0.0229 (10)	0.0236 (10)	0.0324 (12)
C6	0.0256 (11)	0.0365 (12)	0.0348 (12)	0.0106 (9)	0.0138 (9)	0.0235 (10)
C7	0.0234 (10)	0.0275 (10)	0.0213 (10)	0.0105 (8)	0.0112 (8)	0.0132 (9)
C8	0.0187 (9)	0.0235 (10)	0.0208 (10)	0.0035 (8)	0.0067 (8)	0.0113 (8)
C9	0.0198 (9)	0.0214 (10)	0.0181 (10)	0.0034 (8)	0.0056 (8)	0.0055 (8)
C10	0.0334 (12)	0.0255 (11)	0.0309 (12)	0.0068 (9)	0.0163 (10)	0.0113 (9)
C11	0.0423 (13)	0.0239 (11)	0.0400 (14)	0.0113 (10)	0.0199 (11)	0.0085 (10)
C12	0.0317 (12)	0.0366 (13)	0.0305 (12)	0.0101 (10)	0.0167 (10)	0.0041 (10)
C13	0.0254 (11)	0.0409 (12)	0.0227 (11)	0.0036 (9)	0.0114 (9)	0.0104 (9)
C14	0.0235 (10)	0.0277 (11)	0.0213 (10)	0.0048 (8)	0.0070 (8)	0.0103 (9)
C15	0.0292 (11)	0.0263 (11)	0.0451 (14)	0.0039 (9)	0.0081 (10)	0.0186 (11)

### Geometric parameters (Å, °)

**Table:**

S—O2	1.492 (2)	C6—H6	0.9500
S—C1	1.769 (2)	C8—C9	1.461 (3)
S—C15	1.792 (2)	C9—C14	1.396 (3)
O1—C7	1.384 (2)	C9—C10	1.400 (3)
O1—C8	1.384 (2)	C10—C11	1.382 (3)
C1—C8	1.358 (3)	C10—H10	0.9500
C1—C2	1.449 (3)	C11—C12	1.386 (3)
C2—C7	1.394 (3)	C11—H11	0.9500
C2—C3	1.398 (3)	C12—C13	1.382 (3)
C3—C4	1.381 (3)	C12—H12	0.9500
C3—H3	0.9500	C13—C14	1.389 (3)
C4—C5	1.394 (3)	C13—H13	0.9500
C4—H4	0.9500	C14—H14	0.9500
C5—C6	1.383 (3)	C15—H15A	0.9800
C5—H5	0.9500	C15—H15B	0.9800
C6—C7	1.383 (3)	C15—H15C	0.9800
			
O2—S—C1	106.42 (9)	C1—C8—C9	134.62 (17)
O2—S—C15	107.19 (10)	O1—C8—C9	114.80 (16)
C1—S—C15	98.28 (10)	C14—C9—C10	118.74 (18)
C7—O1—C8	106.40 (14)	C14—C9—C8	121.25 (17)
C8—C1—C2	107.57 (17)	C10—C9—C8	119.95 (18)
C8—C1—S	125.35 (15)	C11—C10—C9	120.4 (2)
C2—C1—S	126.67 (14)	C11—C10—H10	119.8
C7—C2—C3	118.79 (18)	C9—C10—H10	119.8
C7—C2—C1	104.81 (16)	C10—C11—C12	120.3 (2)
C3—C2—C1	136.38 (19)	C10—C11—H11	119.8
C4—C3—C2	117.8 (2)	C12—C11—H11	119.8
C4—C3—H3	121.1	C13—C12—C11	119.9 (2)
C2—C3—H3	121.1	C13—C12—H12	120.1
C3—C4—C5	121.8 (2)	C11—C12—H12	120.1
C3—C4—H4	119.1	C12—C13—C14	120.2 (2)
C5—C4—H4	119.1	C12—C13—H13	119.9
C6—C5—C4	121.76 (19)	C14—C13—H13	119.9
C6—C5—H5	119.1	C13—C14—C9	120.40 (19)
C4—C5—H5	119.1	C13—C14—H14	119.8
C7—C6—C5	115.5 (2)	C9—C14—H14	119.8
C7—C6—H6	122.3	S—C15—H15A	109.5
C5—C6—H6	122.3	S—C15—H15B	109.5
C6—C7—O1	125.00 (18)	H15A—C15—H15B	109.5
C6—C7—C2	124.38 (18)	S—C15—H15C	109.5
O1—C7—C2	110.62 (16)	H15A—C15—H15C	109.5
C1—C8—O1	110.58 (16)	H15B—C15—H15C	109.5
			
O2—S—C1—C8	−130.84 (18)	C1—C2—C7—O1	0.4 (2)
C15—S—C1—C8	118.42 (19)	C2—C1—C8—O1	1.2 (2)
O2—S—C1—C2	40.85 (19)	S—C1—C8—O1	174.25 (13)
C15—S—C1—C2	−69.89 (19)	C2—C1—C8—C9	−178.8 (2)
C8—C1—C2—C7	−1.0 (2)	S—C1—C8—C9	−5.8 (3)
S—C1—C2—C7	−173.87 (15)	C7—O1—C8—C1	−1.0 (2)
C8—C1—C2—C3	177.6 (2)	C7—O1—C8—C9	179.06 (16)
S—C1—C2—C3	4.7 (3)	C1—C8—C9—C14	−38.8 (3)
C7—C2—C3—C4	−1.6 (3)	O1—C8—C9—C14	141.18 (18)
C1—C2—C3—C4	179.9 (2)	C1—C8—C9—C10	144.1 (2)
C2—C3—C4—C5	0.8 (3)	O1—C8—C9—C10	−36.0 (3)
C3—C4—C5—C6	0.3 (3)	C14—C9—C10—C11	0.0 (3)
C4—C5—C6—C7	−0.4 (3)	C8—C9—C10—C11	177.18 (19)
C5—C6—C7—O1	179.56 (18)	C9—C10—C11—C12	−0.3 (3)
C5—C6—C7—C2	−0.5 (3)	C10—C11—C12—C13	0.7 (4)
C8—O1—C7—C6	−179.69 (19)	C11—C12—C13—C14	−0.9 (3)
C8—O1—C7—C2	0.3 (2)	C12—C13—C14—C9	0.5 (3)
C3—C2—C7—C6	1.5 (3)	C10—C9—C14—C13	−0.1 (3)
C1—C2—C7—C6	−179.59 (19)	C8—C9—C14—C13	−177.25 (18)
C3—C2—C7—O1	−178.49 (16)		

### Hydrogen-bond geometry (Å, °)

**Table:**

*D*—H···*A*	*D*—H	H···*A*	*D*···*A*	*D*—H···*A*
C15—H15C···O2^i^	0.98	2.34	3.290 (3)	164

Symmetry codes: (i) −*x*+1, −*y*+1, −*z*+1.
